# Evolutionary features of 8K (KDa) silencing suppressor protein of Potato mop-top virus

**DOI:** 10.22099/mbrc.2018.28458.1304

**Published:** 2018-03

**Authors:** Aminallah Tahmasebi, Alireza Afsharifar, Ahmad Heydari, Mohammad Mehrabadi

**Affiliations:** 1 Plant Virology Research Center, College of Agriculture, Shiraz University, Shiraz, Iran; 2 Firoozabad Center for Higher Education, Iran; 3 Department of Entomology, Faculty of Agriculture, Tarbiat Modares University, Tehran, Iran

**Keywords:** Genetic variation, 8K, Potato mop-top virus, RNA silencing suppressor

## Abstract

The cysteine-rich 8K protein of Potato mop-top virus (PMTV) suppresses host RNA silencing. In this study, evolutionary analysis of 8K sequences of PMTV isolates was studied on the basis of nucleotide and amino acid sequences. Twenty-one positively selected sites were identified in 8K coding regions. Recombination events were found in the 8K of PMTV isolates with a rate of 1.8. Totally 30 haplotypes were detected with haplotype diversity ranging from 0.8 to 1.0 and nucleotide diversity from 7.58 to 13.62. The positions 33 and 30 indicated the highly positive and negative selection (with the highest and the lowest dN-dS values), respectively. Tajima’s D test suggested that 8K is evolving with a strong positive selection for worldwide isolates. High frequency of segregating sites was identified along 204 positions of *8K*. Moreover, in this study, we used Shannon entropy-based approach to evaluate the variability of each site of nucleotide and corresponding amino acid. Based on Shannon entropy method, 139 and 97 nucleotide sites had the highest entropy value, while 47 and 33 amino acid sites showed the most diversity along 8K sequences. Our findings suggest that 8K as an RNA silencing suppressor evolves rapidly. Taken together, its variability might play a big threat to infect other plants or overcome resistant cultivars.

## INTRODUCTION

Potato mop-top virus** (**PMTV) belongs to the *Pomovirus *genus in the *Virgaviridae* family [1]. PMTV occurs in many potato-production areas of the world. PMTV has been detected from many parts of the world including Americas, parts of Asia and Europe [2-4]. The particles of PMTV are tubular rod-shaped with a tripartite, positive-sense and single-stranded RNA genome [5]. The tripartite genome of PMTV contains eight open reading frames (ORFs). RNA1 and RNA2 encode virus replication and encapsidation/transmission functions, respectively [6, 7]. A triple gene block (TGB) and 8K are encoded by RNA3 [5]. TGB-encoded proteins cause the cell-to-cell movement of PMTV [8, 9]. The fourth protein, 8K increases the virulence of the virus and acts as an RNA silencing suppressor [10]. 

PMTV causes brown arcs and circles in potato tubers, thereby it is considered as a big threat to make the potato tubers unmarketable [2, 3]. PMTV isolates from Europe, North America and Asia share a high level of sequence identity [11-16]. Viral pathogenicity has often been related to viral suppressors of RNA silencing (VSR) [17, 18]. VSR proteins have been evolved by viruses to counter RNA silencing based-defense. RNA silencing acts as a potent antiviral defense mechanism to protect plants against viruses [19]. It was shown that the relative rate of VSRs evolution is faster than other virus genes [20]. PMTV *RdRp*, *CP*, *CP-RT* and *TGB* genes showed negative selection, while the *8K* was under positive selection. Also, the *8K* sequence of the PMTV isolates indicated the lowest identity among PMTV genes [21]. However, there is little information about evolutionary features of 8K as a VSR in worldwide isolates. Therefore, the evolutionary analysis of 8K suppressor is essential to develop better strategies for controlling PMTV infection. 

In the present study, the complexity of 8K was characterized using two indices: the mean nucleotide diversity and the Shannon entropy. Shannon entropy was originally developed by Claude Shannon and it is also used to measure the information content or complexity of a series. Shannon information is regularly used to measure species diversity and population genetics [22, 23]. Also, the mean nucleotide diversity measures the average number of nucleotide differences between any two sequences [24].

## MATERIALS AND METHODS

**Multiple-sequence alignment: **Multiple-sequence alignment was performed among worldwide isolates using CLC Main Workbench 7.7.2 software. A number of 72 sequences of 8K were then aligned, concatenating triplets according to the sequence alignment using the MEGA program, version 6.06. The model of nucleotide or amino acid substitutions was evaluated by a maximum likelihood approach. Substitution pattern and rates were estimated with the different models. 

Non-synonymous (dN) and synonymous (dS) substitution rates are estimated using the joint Maximum Likelihood reconstructions under a Muse-Gaut model of codon substitution and Felsenstein model of nucleotide substitution. Mean (relative) evolutionary rate was estimated using the Jones-Taylor-Thornton model (+G). A discrete Gamma (+G) distribution was applied to model evolutionary rate differences among sites. The substitution patterns (transitional and transversional substitution rates) and nucleotide frequencies for 8K gene were estimated based on the Tamura-Nei model. 

**Comparative analysis of 8K sequence evolution: **To identify specific amino acid sites under selective constraints, the number of differences between dN and dS substitution rates were estimated for each position in the alignments using the MEGA program, version 6.06. A dN-dS value of > 0 is considered as evidence for positive selection, while values of < 0 are indicative of negative selection. The statistical tests for genetic differentiation, estimation of a number of haplotypes and haplotype diversity was performed using DnaSP 5.10.01 software [25]. Potential recombination events between nucleotide sequences of 8K sequences were assayed using DnaSP 5.10.01 software. 

**Entropy analysis: **Entropy analysis was used to evaluate the variability and complexity of each site of nucleotide and amino acid. Shannon entropy was calculated using the definition [23]: $H_{i}=-\sum_{j}p_{\mathrm{ij}}\log_{2}p_{\mathrm{ij}},\mathrm{for each site i}$, where $p_{\mathrm{ij}}$ is the frequency of nucleotide or amino acid j in the site i and $H_{i}$ is the entropy of each site i. Index j is equal to 1,2,3 or 4 corresponding to nucleotides A , C, G and T respectively or an integer among 1,2,…,20 corresponding to different codons of amino acids alphabet. Sites with entropy value equal to 0 are defined as those which are without complexity and diversity.

## Results

**Figure 1:**
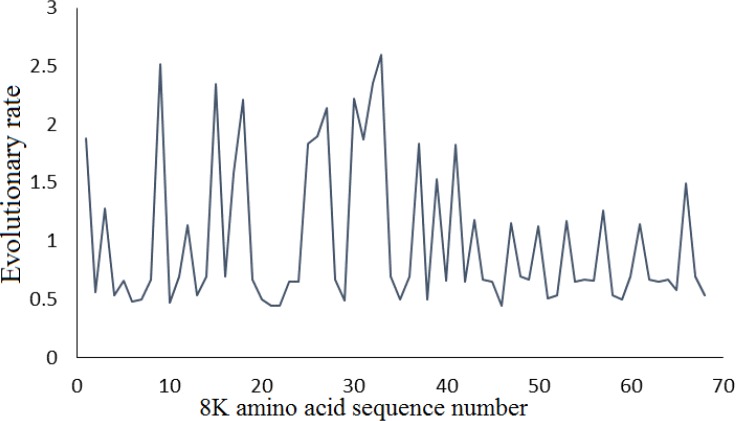
Mean (relative) evolutionary rates over 8K amino acid sequence. Mean (relative) evolutionary rates are shown for each site next to the site number. The average of evolutionary rate across all sites is 1. This means that sites showing a rate <1 are evolving more slowly than average whereas those with a rate>1 are evolving faster than average. The analysis involved 72 amino acid sequences

The substitution pattern for *8K* gene based on the Tamura-Nei model showed that the transitional substitution rates were from 8.69 to 28.06, while transversional substitution rates varied from 0.93 to 3.02. Compared to transversional substitutions, high transitional substitutions were identified along the *8K* region. The nucleotide frequencies of *8K* gene were: 21.61% (A), 41.58% (T/U), 12.87% (C), and 23.94% (G). The ratios of transition and transversion were k1= 14.747 (purines) and k2= 9.301 (pyrimidines). The transition/ transversion bias was R= 5.083 and R= [A*G*k1 +T*C*k2]/[(A+G) *(T+C)]. Mean evolutionary rates were indicated for each 8K amino acid position (Fig. 1).

**Figure 2:**
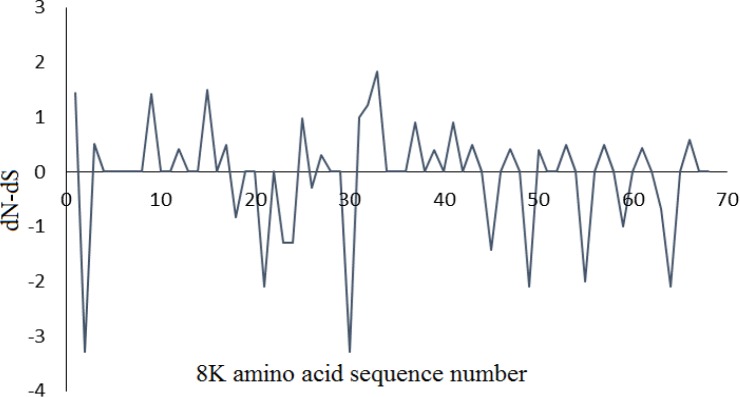
The dN-dS along 72 samples of 8K amino acid sequences. This ratio is achieved by differences between nonsynonymous and synonymous positions (dN-dS). The dN-dS index below zero is consistent with negative selection against protein change. In contrast, a dN-dS index above zero may be an indication that adaptive or positive selection is driving gene divergence. dS and dN are the numbers of synonymous and nonsynonymous substitutions per site, respectively

The average evolutionary rate across all positions is considered 1; a rate < 1 shows that site evolves slower than the average while the sites with a rate> 1 evolve faster than the average. These rates were estimated using the Jones-Taylor-Thornton model (+G) [26]. The differences in evolutionary rate among sites were based on a discrete Gamma (+G) distribution model. The ML estimate of the gamma parameter was 0.7798. The maximum Log likelihood for this computation was -464.203. Recombination events were identified in the 8K of PMTV isolates with average value; 1.8. Seven recombination sites were detected between nucleotides of (1,25); (25,43); (74,80); (80,89); (89,90); (95,121); (121,139). The degree of negative selection of genes can be estimated by Tajima’s D and dN-dS approaches. Tajima’s D method computes the differences between the number of segregating sites and neutral expectation. Tajima’s D test suggested that 8K is evolving with a strong positive selection for worldwide isolates. The dN-dS approach evaluated the selection by differences between nonsynonymous and synonymous positions (dN-dS) of *8K *sequences. The dN-dS value below zero is representing negative selection. Whereas, a dN-dS value above zero is considered as positive selection. In this study, the mean of dN-dS values showed heterogeneous dN-dS values (from -3.29 to 1.82). The positions 33 and 30 indicated the highly positive and negative selection (with the highest and the lowest dN-dS values), respectively (Fig. 2). 

**Figure 3:**
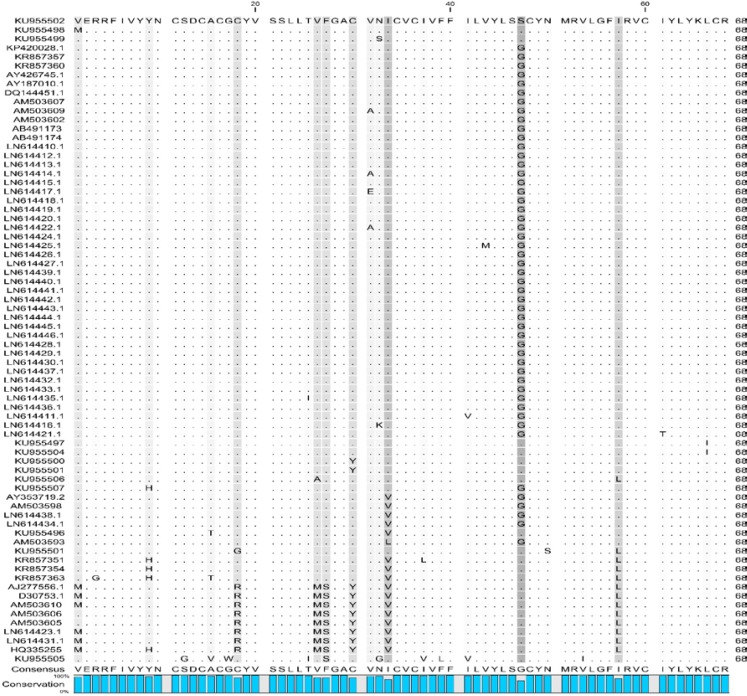
Multiple alignment of the amino-acid sequences of PMTV 8K suppressors from the worldwide isolates

Totally, a number of 21 positively selected sites were identified using site-by-site analysis of the dN/dS ratio. Also, 30 haplotypes were detected and haplotype diversity varied from 0.8 to 1.0 and nucleotide diversity from 7.58 to 13.62. High frequency of segregating sites (42 segregating sites and more than 20.59 % of the total number of sequence bases) were identified along 204 positions of *8K* (Table 1). Alignment of 8K sequences identified some amino acid substitutions among them. A change was found in the start codon of 8K in some isolates. The replacement of the AUG start codon by the GUG occurred. 9.72 percent of the isolates uses AUG (methionine amino acid) as a translation initiation codon, while GUG (valine amino acid) is replaced in other isolates (90.28 %) (Fig. 3).

**Figure 4:**
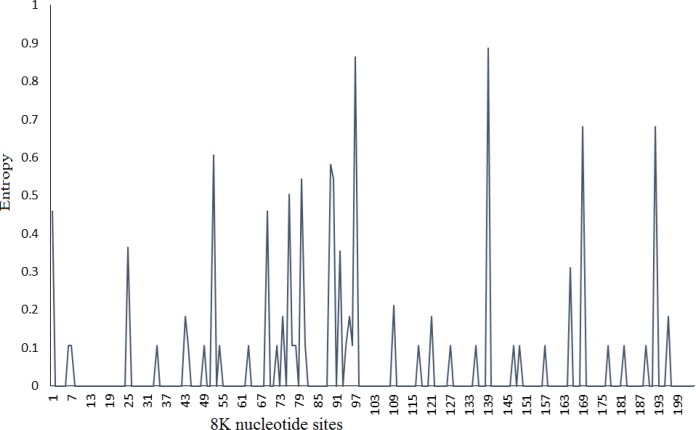
Shannon entropy for 8K nucleotide sites from 72 worldwide sequences. Entropy was calculated for each nucleotide position along 8K

**Figure 5:**
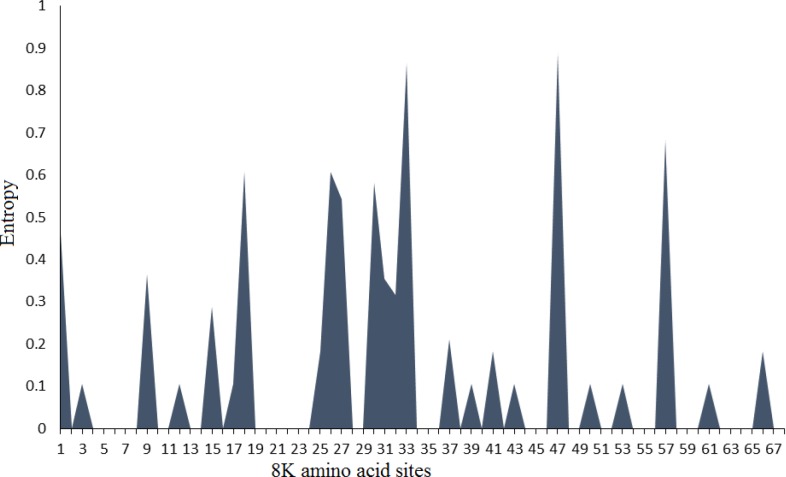
Histograms of amino acid positions occurring at different entropy values in 8K genetic diversity study

In this study, we used Shannon entropy-based approach to evaluate the variability of each site of nucleotide and corresponding amino acid. Based on Shannon entropy method, 139 and 97 nucleotide sites had the highest entropy value, while 47 and 33 amino acid sites showed the most diversity along 8K sequences (Figs. 4, 5).

## DISCUSSION

RNA viruses are faced with different host antiviral responses including RNA silencing that performs sequence-specific inhibition of viral mRNAs expression. To counteract the host RNA silencing, viruses develop VSRs such as the 8K protein of PMTV. In this study, the complexity of 8K nucleotide and amino acid sites was characterized. Position 33 indicated the highest evolutionary rate based on the mean evolutionary rate for each site, which was further confirmed by Shannon entropy approach (with a high-value complexity) and strongly positive selection. The evolutionary rate results were confirmed by the *dN-dS *index, where a strongly positive selection was observed on position 33. Therefore, this amino acid evolves more than those in the other positions. Furthermore, the most positive pressure on position 33 shows its importance for virus survival and flexibility in response to evolutionary forces. While, the lowest evolutionary rates were related to the amino acid positions 21, 22 and 46. We assume that these amino acid sites can play an important role in 8K protein function. The results derived here suggested that Shannon’s information entropy can be used as a measure to evaluate the genetic diversity of viruses and their evolution [22, 23]. However, there is increasing tendency to incorporate entropy methods into population genetic analysis that it might be extended to virus diversity and evolution. Twenty-one positively selected sites were observed during the 8K sequence, which represents that the *8K* gene is under strong positive selection suggesting the acceleration in the divergence of the *8K* gene. Recently, positive selection on another RNA silencing suppressor, P1 of Rice yellow mottle virus has been reported [27]. Thus, the increased rate of VSRs evolution might be due to reduced constraint than other viral genes with highly conserved patterns [20]. Moreover, our results showed that the AUG start codon can be replaced by GUG in *8K* of PMTV isolates. The utilization of initiating non-AUG codon for maintenance of proper ratios of proteins has been reported for both cellular and viral mRNAs [28], including plant viruses [29]. The increase of GUG start codon in PMTV isolates can lead to change in expression level of 8K protein. Further studies would be concentrated on assessing the impact of AUG and GUG start codons on the virulence of the virus. 

Point mutations on VSRs, including the helper component proteinase (HC-Pro), affect their suppression function [30-32]. Also, mutations on sites under positive selection can enhance the strength of 8K suppressor. Another important mechanism of the 8K evolution and virus genetic variation is recombination, which can also play a key role in maintaining the effectiveness of purifying selection by preventing the accumulation of deleterious mutations [33]. The high number of sites positively selected and recombination events suggest that 8K suppressor evolves rapidly. Also, negative Tajima’s D showed demographic forces and expansion on PMTV population. Thus, the variability and acceleration in the divergence of the *8K* gene might be a virus strategy to constitute an adaptation to the different plant hosts in various agro-systems during PMTV evolution. The rapid evolution of the PMTV 8K could be due to the evolutionary conflict between the RNA silencing pathway and its suppression to increase virus replication and spread [34]. 

 In this study, we determined the high evolutionary rate of 8K sequences using bioinformatics tools. Considering the role of 8K in enhancing the virulence of PMTV and also as an RNA silencing suppressor of plants, its variability might be possible to play a big threat to infect other plants or overcome resistant cultivars.
